# Supplementary material for: Benchmarking spike source localization algorithms in high density probes
Source: PLoS Comput Biol. 2026 Mar 16;22(3):e1014059. doi: 10.1371/journal.pcbi.1014059 (PMC13002180; doi:10.1371/journal.pcbi.1014059)
Supplement: Attachment — Submitted filename: 2025-09-01_PlosReview_localisation.pdf [file pcbi.1014059.s001.pdf]

Summary

The authors present the result of a benchmarking study regarding the localisation of spiking signals recorded on multi-electrode arrays (MEAs).

They compare 3 localisation methods: center of mass (CoM), grid convolution (GC) and monopolar triangulation (MT). To do so they use simulated neurons of the somatosensory rat cortex using the NEURON software, as well as a Neuropixel 1 dataset that simultaneously record units with intracellular recordings (using patch-clamping) in the same brain areas.

To simulate long-term chronic implantation scenarios, the authors simulated electrode degradation by replacing channels with noise and compared the performance of the 3 methods on this degraded data.

The results show high accuracy triangulation and grid convolution on simulated data versus center of mass. When moving to a real dataset, the results show the conservative CoM having a higher accuracy than GC, then MT, further confirmed when adding electrode degradation. The authors conclude that conservative estimates are more robust when planning long-term recordings from chronic implants.

Key strengths and limitations

This is a very welcome and needed initiative to try and deconstruct the “spike sorting black box” in smaller, tractable modules to evaluate them thoroughly. The overall methodology of first using synthetic data, then using the Marques-Smith dataset with Neuropixel / patch-clamp simultaneously indeed lays the foundation for a solid benchmark for spike localisation. The analysis of localisation algorithms is then well-conducted and results are presented in a straightforward manner.

The main shortcoming of the paper is the lack of attention to essential aspects of MEAs data analysis. First pre-processing of whole probe data and denoising of the waveforms is not mentioned. Second the dependence of the results on the geometry and the associated meta-parameters of each localiser is also not explored. In the simulation of electrode degradation, authors do not take into account that it is now standard to perform automatic anomalous channel detection and removal/interpolation before any analysis, real-time or not.

In a nutshell pre-processing will drastically change any spike sorter results, and this study is no different in this regard. Since previous work in the field uses heavy denoising and pre-processing before applying their localisation algorithms - both at the whole probe and single waveform level - this benchmark needs to be revised in this direction to yield useful recommendations for practitioners that would also use those methods.

Specific feedback

a) The paper methods are scarce, specifically on the following aspects:

- Pre-processing:
  - what pre-processing is applied to the Neuropixel data ?
  - how were the waveforms extracted and what further denoising is applied to the waveforms ?
  - how was the data spike sorted for the units used in the localisation ?
  - how were the templates formed for the synthetic data ?
- Localisation meta-parameters: both GC and MT use meta-parameters, such as size of the grid and radius of channels. What are their values? Was any parameter search conducted to assess the robustness of the parameters relative to degradation ?
- Figure 3: how is the drift estimated ?
- electrode degradation: what is the noise level added ? Are those supposed to mimic dead channels or noisy channels ?

b) The noise added to simulation is arbitrary (1uV Gaussian). Neuropixel probes will yield on the order of 5-10 uV rms noise. Computing templates by averaging spikes yields an attenuation of noise of the order of sqrt(n_summed_spikes), a factor of around 1/23 for 500 summed spikes (cf. uncertainty propagation). So it seems the noise is too high for the template case, and too low for the single spike case.

c) Why is the geometry of simulations different from a Neuropixel probe used in the real data ? Exploring geometry differences could be out-of-scope for this benchmark, and authors could focus only on the differences simulated versus acquired. Or authors could explore geometry differences using different simulations. Either case is fine, but at least one of the simulations should share the same geometry as the experimental data.

d) Running times do not seem representative: in figure 2D the runtime of the GC method is only slightly higher transitioning from templates to spikes. It seems there is an overhead in computing the convolution grid for each template that doesn’t happen in the single spikes. It is probable that anyone wanting to use the technique real-time would pre-compute the convolution stencils for a given probe geometry, and this is the running time that would be informative for a practitioner.

e) More attention should be given to the data preparation and access to claim having provided a benchmark. Providing a github repository is not enough: here I recommend to truly go the extra mile of preparing intermediate datasets such as spike trains and waveforms, along with their associated localisation values (no need to reproduce the already published raw data).

Minor comments

- Summary: define electrode decay when first mentioning it. Settle for decay or degradation throughout the paper (degradation is more explicit IMO)
- Figure 1 B: link each prediction to ground truth with a small line. Put scale.

- Figure 2:
    - A and B: put scales
    - C: and D: put running time from template to spikes at the same y axis scale, this is hard to read
- Future direction: at least one earlier triangulation application is in seismology as early as 1912 https://www.nature.com/articles/090003b0 !

Conclusion

I find the work here potentially impactful and the comparison protocol elegant, but the paper needs to build more on the domain knowledge of pre-processing to get results more useful to experts. It also needs to disclose the methods more thoroughly.

This work is genuinely a *"first-of-its-kind benchmarking of commonly used neuron localisation algorithms*". The associated annotated benchmark dataset would also be a novelty, and a nice contribution to the community.

As such I would be fully supportive of a revised version, and enjoin the authors on submitting again !
