# Supplementary material for: Benchmarking spike source localization algorithms in high density probes
Source: PLoS Comput Biol. 2026 Mar 16;22(3):e1014059. doi: 10.1371/journal.pcbi.1014059 (PMC13002180; doi:10.1371/journal.pcbi.1014059)
Supplement: Attachment — Submitted filename: Response to Reviewer (2025.12.14).pdf [file pcbi.1014059.s002.pdf]

<u>Response to Reviewer #1 Comments:</u>

General Comment: This is a very welcome and needed initiative to try and deconstruct the "spike sorting black box" in smaller, tractable modules to evaluate them thoroughly. The overall methodology of first using synthetic data, then using the Marques-Smith dataset with Neuropixel / patch-clamp simultaneously indeed lays the foundation for a solid benchmark for spike localisation. The analysis of localisation algorithms is then well-conducted and results are presented in a straightforward manner.

The main shortcoming of the paper is the lack of attention to essential aspects of MEAs data analysis. First pre-processing of whole probe data and denoising of the waveforms is not mentioned. Second the dependence of the results on the geometry and the associated meta-parameters of each localiser is also not explored. In the simulation of electrode degradation, authors do not take into account that it is now standard to perform automatic anomalous channel detection and removal/interpolation before any analysis, real-time or not.

In a nutshell pre-processing will drastically change any spike sorter results, and this study is no different in this regard. Since previous work in the field uses heavy denoising and pre-processing before applying their localisation algorithms - both at the whole probe and single waveform level - this benchmark needs to be revised in this direction to yield useful recommendations for practitioners that would also use those methods.

Response to General Comment: We sincerely thank the reviewer for their notes regarding the relevance of this area and the value of benchmarking spike localization algorithms against ground-truth data in real-world recording conditions. We fully agree with the reviewer's comments that the original manuscript was lacking detail on the processing pipeline, and we have extensively revised the manuscript to address these and other gaps. These revisions include an expanded "Methods" section; clearer explanations of filtering, common-mode referencing, and anomalous channel detection; and a revised simulated dataset matching the Neuropixel 384-channel probe used in the experimental dataset, among other revisions. We believe these changes meaningfully strengthen the rigor and relevance of this paper, and appreciate the reviewer's time on this manuscript.

Comment #1a: Pre-processing: What pre-processing is applied to the Neuropixel data? How were the waveforms extracted and what further denoising is applied to the waveforms? How was the data spike sorted for the units used in the localization? How were the templates formed for the synthetic data?

Response to Comment #1a: We thank the reviewer for this suggestion.

Our pre-processing pipeline consists of three steps. The first step is bandpass filtering from 300 – 3,000 Hz to remove low-frequency artifacts (e.g., local field potentials, low-frequency biological activity such as breathing and heartbeat) and high-frequency noise (e.g., amplifier noise and ADC quantization noise), while maintaining the 300 – 3,000 Hz zone where spike signal is expected. The second step is common median referencing to remove common-mode signals across channels. The third step is anomalous channel detection, using the established protocol from IBL, detecting anomalous channels via coherence (i.e., adjacent channels within brain tissue should have a high degree of signal correlation) and power distribution (i.e., healthy channels should have a normal power distribution across frequencies)[1].

For our benchmarking, we identified simulated (MEArec) and experimental (Marques-Smith) ground-truth datasets where the ground-truth spike trains are known. For the MEArec dataset, we know the underlying spike trains that are used to generate the simulation.[2] For the Marques-Smith dataset, the Neuropixel data is obtained in parallel with patch-clamp recording from the same neuron, where the patch-clamp data, following basic peak detection, provides the ground-truth spike train for the Neuropixel data.[3] As such, we are able to use ground-truth spike trains to extract spikes from de-noised recordings without relying on spike sorting algorithms. Leveraging the known ground-truth spike trains allows us to avoid introducing localization bias from sorting algorithms (as sorters generally rely on some assumption about spike localization) and reduces noise (e.g., spikes not meeting detection thresholds, clustering errors, etc.) that allow us to isolate our analysis to the basic spike localization problem.

For each neuron, we collect short segments of the preprocessed signal centered on each ground-truth spike, using a fixed temporal window that captures the full spike waveform and its immediate context. Waveforms are then aligned in time to the negative peak, to reduce temporal jitter and to isolate the characteristic shape of each neuron's extracellular signature. We then extract template waveforms by aggregating all spikes from the same neuron, in order to obtain a representative waveform that captures the temporal and spatial footprint of the neuron in a given trial.

Revisions to Manuscript: We include the following in "Methods".

Pre-Processing: A standard spike sorting pre-processing pipeline for denoising and anomalous channel detection was applied to both the simulated and experimental datasets. A 300 - 3,000 Hz Butterworth bandpass filter was applied to the raw recordings, followed by common median referencing. We then perform anomalous channel detection using the established IBL protocol, detecting anomalous channels via coherence (i.e., adjacent channels within brain tissue should have a high degree of signal correlation) and power distribution (i.e., healthy channels should have a normal power distribution across frequencies).

Spike and Template Extraction: Following pre-processing of the extracellular recordings, we use the ground-truth spike trains to extract spike waveforms without relying on a spike sorting algorithm. Leveraging the known ground-truth spike trains allows us to avoid bias which would be introduced by spike sorting (which generally relies on some assumption about spike localization) and reduces noise (e.g., detection thresholds, clustering errors, or missed events), and so isolates our analysis to the basic spike localization problem. For each neuron, we collect short segments of the preprocessed signal centered on each ground-truth spike, using a fixed temporal window so that the full spike waveform and its immediate context are captured. Waveforms are then aligned to the peak, to reduce temporal jitter and to isolate the characteristic shape of each neuron's extracellular signature. We then extract template waveforms by aggregating all spikes from the same neuron, in order to obtain a representative waveform that captures the temporal and spatial footprint of the neuron in a given trial.

Comment #1b: Localisation meta-parameters: Both GC and MT use meta-parameters, such as size of the grid and radius of channels. What are their values? Was any parameter search conducted to assess the robustness of the parameters relative to degradation?

Response to Comment #1b: We thank the reviewer for noting the lack of specified meta-parameters, which are important to ensure reproducibility and better understand the characteristics of the spike localization algorithms in the study.

We note that the purpose of this benchmarking study is to evaluate the canonical and most widely used forms of these algorithms; indeed, tuning algorithms to this dataset may result in "overfitting" the more expressive algorithms to our two datasets. As such, we did not conduct a parameter search for those which maximize the robustness to degradation. We acknowledge that solving for optimal parameters could be an interesting area for further study, especially as it relates to biologically-linked parameters (e.g., signal decay).

For monopolar triangulation meta-parameters, we use (i) a least squares optimizer, (ii) a local radius of 75 µm around the peak channel to select channels that contribute to the optimization problem, (iii) a search space constrained to a 1,000 µm radius around the center-of-mass estimate to regularize against mathematically "optimal" solutions that are unfeasibly far from the peak channel, and (iv) the remaining default parameters in the commonly-used SpikeInterface implementation, and which are publicly available.[4]

For grid convolution meta-parameters, we use (i) a search space of radius 40 µm around the peak channel for an up-sampled grid, (ii) a 5 µm resolution for the up-sampled grid, and (iii) the remaining default parameters in the commonly-used SpikeInterface implementation, and which are publicly available.[4]

Revisions to Manuscript: We include the following in "Methods":

[Regarding monopolar triangulation]… *We use the SpikeInterface implementation of this method, which uses a least-squares optimizer and several regularization parameters including limiting contributing channels to a local radius around the peak channel and limiting the location estimate to a candidate window around the center of mass estimate. We utilize SpikeInterface's default parameters (e.g., local radius of 75µm, candidate window of 1,000µm; please refer to source documentation for full parameter detail). We note that removing regularization negatively impacted MT performance; while this better captures the mathematical properties of MT, we believe that the inclusion of regularization better reflects a real-world, practical implementation of the algorithm.*

[Regarding grid convolution]… *We use the SpikeInterface implementation of this method, which includes regularization parameters including limiting the grid to a local radius around the peak channel. We utilize SpikeInterface's default parameters (e.g., local radius of 40µm, up-sampled grid resolution of 5µm; please refer to source documentation for full parameter detail). We note that removing regularization negatively impacted GC performance, though is not as impactful as it is to MT; while this better captures the mathematical properties of GC, we believe that the inclusion of regularization better reflects a real-world, practical implementation of the algorithm.*

Comment #1c: Figure 3: How is the drift estimated?

Response to Comment #1c: We thank the reviewer for pointing out that the original manuscript did not sufficiently define drift. Drift in Figure 3 and Figure 4 is intended to measure how unstable the localization algorithm is under electrode degradation. Specifically, drift is calculated as the Euclidean distance between (i) the estimated template location under no electrode degradation and (ii) the estimated template location under a given amount of electrode degradation. Among other qualities, one desirable quality of a localization algorithm is that it would robust under electrode degradation; i.e., that as electrodes drop out, the localization estimates do not swing too wildly.

Revisions to Manuscript: We include the following in the Figure 3 and Figure 4 captions:

Estimated drift is the Euclidean distance between the estimated template location without degradation v. the estimated template location with degradation; i.e., a measure of how unstable the localization algorithm is against degradation.

We include the following in “Results”:

Estimated drift is the Euclidean distance between the estimated template location without degradation and the estimated template location with degradation; i.e., a measure of how unstable the localization estimates are against degradation. Stability is important in long-term recordings where we desire to consistently estimate neuron locations over time as electrodes degrade (erroneous drift can result in the same neuron being identified as different neurons over long-term recordings).

Comment #1d: Electrode degradation: What is the noise level added? Are those supposed to mimic dead channels or noisy channels?

Response to Comment #1d: We thank the reviewer for the opportunity to clarify; our intention is to model dead channels rather than mildly noisy channels. Accordingly, we simulate electrode degradation by replacing a proportion of channels with 50 µV uncorrelated Gaussian noise, consistent with failure modes in chronic implants where dead channels output high-amplitude, structureless noise.

Revisions to Manuscript: We include the following in “Methods”:

In order to simulate electrode degradation, we replace a progressively increasing proportion of electrodes with uncorrelated 50µV Gaussian noise, intended to represent channels that lose functionality over a long-term period (e.g., physical damage or disconnection of site, material gliosis causing high impedance around site, etc.).

Comment #2: The noise added to simulation is arbitrary (1uV Gaussian). Neuropixel probes will yield on the order of 5-10 uV rms noise. Computing templates by averaging spikes yields an attenuation of noise of the order of sqrt(n_summed_spikes), a factor of around 1/23 for 500 summed spikes (cf. uncertainty propagation). So it seems the noise is too high for the template case, and too low for the single spike case.

Response to Comment #2: We appreciate this important correction. We apologize for our carelessness in using a 1 μV noise level, which is not representative; we have increased the recording noise in the simulated dataset to 10 μV, and all simulations and figures have been re-run with this updated noise level. As the reviewer notes, this is a more realistic level of recording noise, but that this noise is attenuated when aggregating spikes into templates.

Revisions to Manuscript: We have updated Figure 2 and Figure 3, which we append at the end of this response letter. We include the following in the "Methods" section:

Uncorrelated Gaussian noise with 10 μV standard deviation was then added to the traces.

Comment #3: Why is the geometry of simulations different from a Neuropixel probe used in the real data ? Exploring geometry differences could be out-of-scope for this benchmark, and authors could focus only on the differences simulated versus acquired. Or authors could explore geometry differences using different simulations. Either case is fine, but at least one of the simulations should share the same geometry as the experimental data.

Response to Comment #3: In response to the reviewer's helpful suggestion, we re-ran the simulated dataset using a Neuropixel 384-channel probe geometry identical to that used in the experimental dataset. All figures and results based on the simulated data have been regenerated accordingly.

Revisions to Manuscript: We have updated Figure 2 and Figure 3, which we append at the end of this response letter. We include the following in the "Methods" section:

We use these cell models to simulate a dictionary of biologically plausible templates on a Neuropixel 384-channel probe.

Comment #4: Running times do not seem representative: in figure 2D the runtime of the GC method is only slightly higher transitioning from templates to spikes. It seems there is an overhead in computing the convolution grid for each template that doesn't happen in the single spikes. It is probable that anyone wanting to use the technique real-time would pre-compute the convolution stencils for a given probe geometry, and this is the running time that would be informative for a practitioner.

Response to Comment #4: We thank the reviewer for this comment. After further inspection, the runtimes are less relevant for the experimental dataset since the eleven experimental neurons are captured by different Neuropixel probes; the localization of each neuron therefore requires the instantiation of overhead associated with an entirely new probe, whereas in practice we expect multiple neurons to be picked up by a high-density probe, which amortizes the overhead (which varies meaningfully by algorithm, as the reviewer pointed out).

We have therefore decided to replace the runtime metric with an estimate of median localization error. The localization error in the experimental ground-truth dataset is inflated by lacking knowledge of exact soma location; we know the location of the nearest electrode, which approximates the exact soma location well, but treating the nearest electrode as the "true" location introduces bias equal to distance from nearest electrode to soma. However, after revising the simulated dataset to a matching Neuropixel 384-channel geometry, we realized that the electrode distances in the Neuropixel implies that the maximum distance from the soma to the nearest electrode is less than ~20 μm (for soma within convex hull of electrode array; may be larger if soma is meaningfully outside convex hull, but that is not common in our simulation). The distance between electrodes is therefore reasonably low compared to biological cell morphology and algorithm

localization error, and so we would not expect this bias to dominate "true" localization error, especially at higher levels of electrode degradation.

As such, the median localization error is still informative, particularly at higher degrees of electrode degradation, and we believe more useful than runtime given the limitation the reviewer pointed out. We have revised the manuscript accordingly.

Revisions to Manuscript: We have updated Figure 2 and Figure 4, which we append at the end of this response letter.

Comment #5: More attention should be given to the data preparation and access to claim having provided a benchmark. Providing a github repository is not enough: here I recommend to truly go the extra mile of preparing intermediate datasets such as spike trains and waveforms, along with their associated localisation values (no need to reproduce the already published raw data).

Response to Comment #5: We thank the reviewer for this excellent suggestion. We completely agree and intend this benchmark to be both easily reproducible and re-usable in the development of improved spike localization algorithms, and have made several revisions to this end.

In response to the request to provide the full spike trains, waveforms, and associated localization values, we have provided the full "end to end" pipeline for a simplified dataset. We note that the "end to end" pipeline for the un-simplified simulated dataset to generate the manuscript figures was 4+ terabytes, and so is difficult and expensive to host in a long-term public environment. Our simplified dataset reduces the number of trials and recording lengths, but illustrates the same principles as our full dataset.

Additionally, we have also updated our github repository to be more easily readable and usable. We note that since we seed the randomized algorithms used to generate the simulated dataset as well as used to simulate dead electrodes in the experimental dataset (with the seeds provided in pipeline), our findings are fully reproducible. We have updated the manuscript with the locations of the simplified and full repositories.

Revisions to Manuscript: We have included the following in "Supporting Information":

All analysis scripts and supporting functions used in this study are available at https://github.com/haozhao1996/Spike-Localization-Algorithms, and we have additionally included a simplified simulated dataset including all recordings, spike trains, waveforms, and localization values. The code is released under the MIT License, permitting reuse, modification, and distribution with attribution. The simulated dataset is generated using MEArec (generation pipeline included in repository, and source code publicly available), and the experimental dataset is SPE-1 (publicly available).

Comment #6a: Summary: define electrode decay when first mentioning it. Settle for decay or degradation throughout the paper (degradation is more explicit IMO).

Response to Comment #6a: We thank the reviewer for this recommendation. We agree that "degradation" is more accurate, and so have revised the manuscript to consistently use "electrode degradation" throughout. In its first mention we additionally note that electrode degradation is intended to represent the loss of recording signal from electrode sites on a probe over time, and have added an "Electrode Degradation" sub-section clarifying this further.

Revisions to Manuscript: In its first mention in "Introduction", we include the following defining electrode decay:

While several localization algorithms are currently used, there has been no ground-truth benchmarking of their performance and robustness against degradation (i.e., loss of recording signal from electrode sites on a probe over time).

We include the following in "Methods":

In order to simulate electrode degradation, we replace a progressively increasing proportion of electrodes with uncorrelated 50µV Gaussian noise, intended to represent channels that lose functionality over a long-term period (e.g., physical damage or disconnection of site, material gliosis causing high impedance around site, etc.).

Comment #6b: Figure 1 B: link each prediction to ground truth with a small line. Put scale.

Response to Comment #6b: We thank the reviewer for this helpful suggestion. We have added small linking lines in blue between estimated and ground-truth locations, as well as added a scale bar.

Revisions to Manuscript: We have updated Figure 1, which we append at the end of this response letter. We include the following in the Figure 1 caption:

Simulated electrophysiological data (MEArec), where green dots denote true neuron locations, red dots denote estimated neuron locations, and blue lines connect the true and estimated locations for a given neuron.

Comment #6c: Figure 2: A and B: put scales. C and D: put running time from template to spikes at the same y axis scale, this is hard to read.

Response to Comment #6c: We thank the reviewer for this helpful suggestion. We have conformed the runtimes to the same y-axis, as well as added a scale bar. We have additionally conformed the y-axis for other metrics (e.g., localization error).

Revisions to Manuscript: We have updated Figure 2, which we append at the end of this response letter.

Comment #6d: Future direction: at least one earlier triangulation application is in seismology as early as 1912 https://www.nature.com/articles/090003b0!

Response to Comment #6d: We thank the reviewer for this interesting reference. This is an interesting example of achieving 2-D triangulation using only two points (instead of three) by knowing the azimuth towards the epicenter at each point. We have added mention of early triangulation work in seismology to the "Future Directions" section.

Revisions to Manuscript: We have included the following in "References":

Galitzin B, Walker G. Determination of the Epicentre of an Earthquake. Nature. 1912. doi:10.1038/090003b0

<u>Response to Reviewer #2 Comments:</u>

General Comment: The theme is interesting and important, and the manuscript is decent and technically sound. It is, however, not very thorough, and has room for improvement. Below are a few comments and suggestions.

Response to General Comment: We sincerely thank the reviewer for their notes regarding the relevance of this area and the value of benchmarking spike localization algorithms against ground-truth data in real-world recording conditions. We fully agree with the reviewer's comments that the original manuscript was not sufficiently thorough, and have revised the manuscript extensively to provide additional detail as well as make publicly accessible a complete sample dataset along with experimental pipeline for open examination. These revisions include an expanded "Methods" section; clearer explanations of filtering, common-mode referencing, and anomalous channel detection; and a revised simulated dataset matching the Neuropixel 384-channel probe used in the experimental dataset. We believe these changes meaningfully strengthen the rigor and relevance of this paper, and appreciate the reviewer's time on this manuscript.

Comment #1: The Methods section could be expanded. For example, what database of cell models are used? Are the cell models a mix of pyramidal cells and interneurons?

Response to Comment #1: We thank the reviewer for this question. In our simulations, we use a mix of pyramidal cells and interneurons derived from the Neocortical Microcircuit Collaboration portal.[5,6] These specific models are from layer five of a juvenile rat somatosensory cortex. In our simulation, we use a ratio of seven excitatory to three inhibitory neurons.

Revisions to Manuscript: We have included the following in "Methods":

We use a dictionary of 13 cell models (representing a mix of excitatory and inhibitory neurons) from layer 5 of a juvenile rat somatosensory cortex, provided via the Neocortical Microcircuit Collaboration portal.

Comment #2: It should be relatively simple to emulate the Neuropixel probe in the simulations, instead of using a generic square probe. This would make simulated and experimental verification easier to compare. Do the authors expect the MT to perform worse than GC and COM in this case, or only in the actual experimental data (as stated in Line 191)? One could then also perhaps test the effect of not knowing the exact location of the soma (only the nearest electrode) in the experimental data, since this might also affect conclusions from the localization.

Response to Comment #2: We fully agree with the reviewer's suggestion. In response to the reviewer's helpful suggestion, we re-ran the simulated dataset using a Neuropixel 384-channel probe geometry identical to that used in the experimental dataset. All figures and results based on the simulated data have been regenerated accordingly, ensuring consistency with experimental conditions.

As the reviewer suggests, we additionally test the impact of not knowing the true neuron location, which we expect would add a baseline amount of localization error. We have prepared Response Figure 1, which we have not included in the revised manuscript but include on the following page.

Revisions to Manuscript: We have updated Figure 2 and Figure 3, which we append at the end of this response letter. We have included the following in "Methods":

We use these cell models to simulate a dictionary of biologically plausible templates on a Neuropixel 384-channel probe.

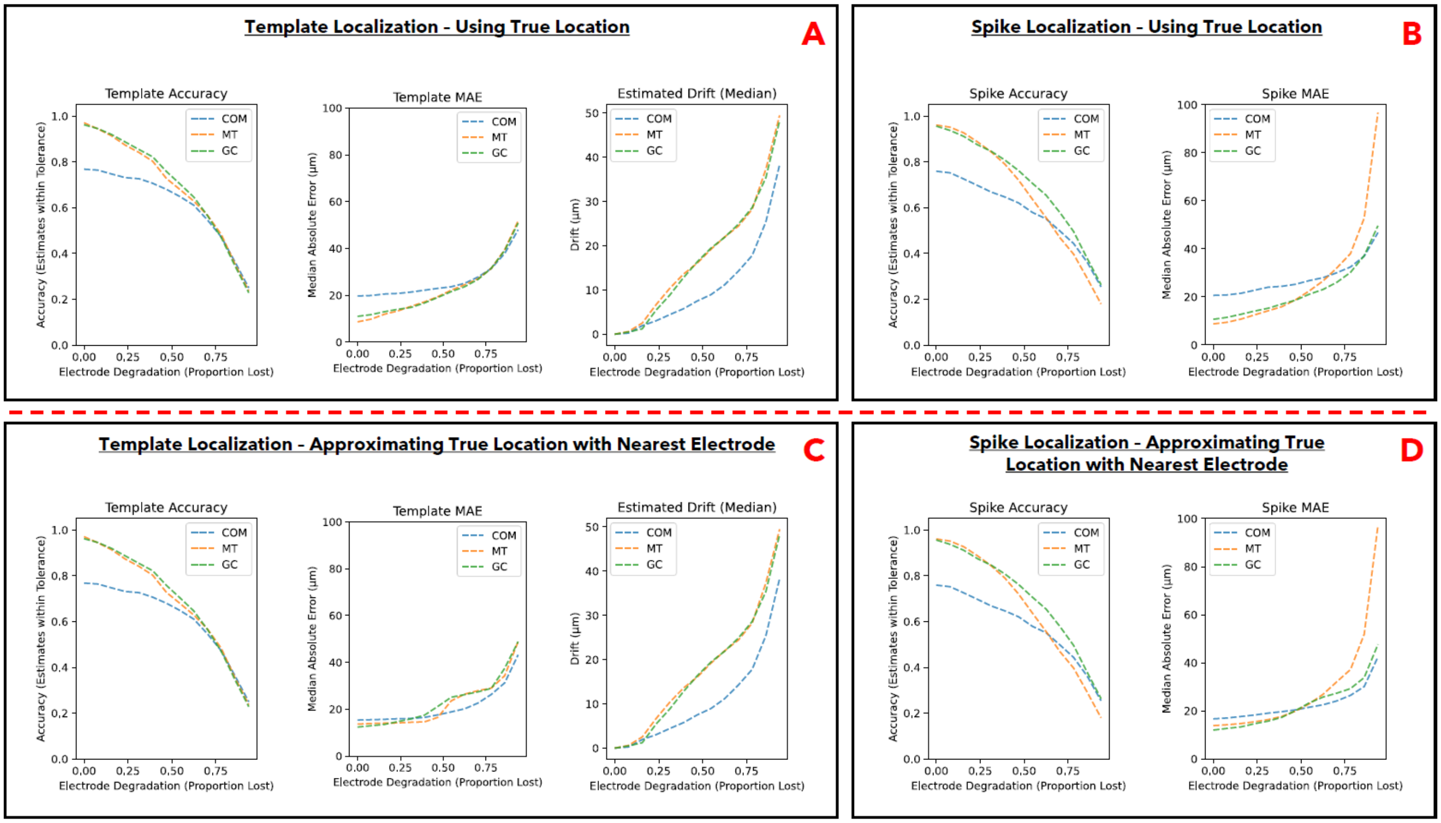

Response Figure 1: Comparison of localization performance on simulated dataset (MEArec) when the true neuron location is approximated with the nearest electrode to the true neuron location (similar to experimental dataset). **A, B:** Localization performance using true neuron location, identical to Fig. 3A and 3B. **C, D:** Localization performance when true neuron location is approximated with the nearest electrode to the true neuron location. We note no change in the accuracy and drift metrics following this approximation. We note the median localization errors did change slightly, but the general trends remain consistent.

Comment #3: Line 58: "Finally, uncorrelated Gaussian noise with 1 mV standard deviation was added to the traces." This number seems extremely high, and is probably wrong. I would have expected the noise level to more like 10 µV?

Response to Comment #3: We appreciate this important correction, and apologize for our carelessness. We have revised the recording noise in the simulated dataset to 10 µV; all simulations and figures have been re-run with this updated noise level, and all text has been corrected as well. As the reviewer notes, this is a more realistic level of recording noise.

Revisions to Manuscript: We have updated Figure 2 and Figure 3, which we append at the end of this response letter. We have included the following in "Methods":

Uncorrelated Gaussian noise with 10 µV standard deviation was then added to the traces.

Comment #4: Line 55: "30 nm apart" Is the unit here wrong perhaps?

Response to Comment #4: We appreciate the reviewer drawing attention to this, and this was indeed a unit error. We have replaced this geometry with a Neuropixel 384-channel probe.

Revisions to Manuscript: We have included the following in "Methods":

The simulated dataset uses the MEArec simulator to generate ground truth recordings using biophysically detailed multicompartment models, and follows similar protocols to previous analyses used to simulate ground truth conditions. We use a dictionary of 13 cell models (representing a mix of excitatory and inhibitory neurons) from layer 5 of a juvenile rat somatosensory cortex, provided via the Neocortical Microcircuit Collaboration portal. We use these cell models to simulate a dictionary of biologically plausible templates on a Neuropixel 384-channel probe.

Comment #5: Line 126: "(Neuropixel 384-channel), which is 5 mm in length and 40 µm in width" Are these numbers correct? They seem different than previous numbers I have seen.

Response to Comment #5: We thank the reviewer for this comment, and we have refined our calculation and revised to 3.832 mm in length and 60 µm in width (approximated in manuscript as roughly 4 mm in length and 60 µm in width). Please note that while the quoted dimensions of Neuropixel are 10mm and 70µm, this includes non-recording area outside of the electrode array, whereas for purposes of this study we are interested solely in dimensions of the electrode array.[7, 8]

Our estimates are based on (i) electrodes of size 12 µm by 12 µm, (ii) four staggered columns spaced 16 µm center-to-center ($12 + 16 \times 3 = 60$ µm width), and (iii) 192 staggered rows spaced 20 µm vertical distance center-to-center ($12 + 20 \times 191 = 3832$ µm length). We note our estimate is in agreement with other estimates; e.g., "The array of 384 adjacent recording sites covers a brain area of approximately 60 µm × 3800 µm (W × L)" (Fiath et al. 2021).[9]

Revisions to Manuscript: We have included the following in "Results":

This limitation is more pronounced in our selected probe geometry (Neuropixel 384-channel probe), where the electrode array is roughly 4mm in length and 60µm in width, and so approximates a "linear" one-dimensional probe.

Comment #6: These two papers might be relevant for the discussion, since they also work on spike localization in high density probes:

Delgado Ruz & Schultz (2014). Localising and classifying neurons from high density MEA recordings. Journal of Neuroscience Methods, 233, 115–128.

Buccino et al (2018). Combining biophysical modeling and deep learning for multi-electrode array neuron localization and classification. Journal of Neurophysiology, 120, 1212–1232.

Response to Comment #6: We appreciate the reviewer's suggestion and have now added both recommended papers—Delgado Ruz & Schultz (2014) and Buccino et al. (2018)—to the Discussion section. These works provide valuable context on high-density MEA localization and complement our triangulation and convolution-based benchmark.

Revisions to Manuscript: We have added the following references:

Ruz ID, Schultz SR. Localising and classifying neurons from high density MEA recordings. Journal of Neuroscience Methods. 2014. doi:10.1016/j.jneumeth.2014.05.037.

Buccino AP, et al. Combining biophysical modeling and deep learning for multi-electrode array neuron localization and classification. Journal of Neurophysiology. 2018. doi:10.1152/jn.00210.2018

References

[1] Laboratory IB. Spike sorting pipeline for the International Brain Laboratory. doi:10.6084/m9.figshare.19705522.v4
[2] Buccino AP, Einevoll GT. MEArec: A fast and customizable testbench simulator for ground-truth extracellular spiking activity. Neuroinform. 2021;19:185–204. doi:10.1007/s12021-020-09467-7.
[3] Marques-Smith A, et al. Recording from the same neuron with high-density CMOS probes and patch-clamp: a ground-truth dataset and an experiment in collaboration. bioRxiv. 2020. doi:10.1101/370080.
[4] Garcia S, Buccino AP, Hurwitz C, et al. SpikeInterface, a unified framework for spike sorting. eLife. 2022;11:e72140. doi:10.7554/eLife.72140.
[5] Markram H, et al. Reconstruction and Simulation of Neocortical Microcircuitry. Cell. 2015. doi:10.1016/j.cell.2015.09.029
[6] Ramaswamy S, Courcol J, Abdellah M, et al. The neocortical microcircuit collaboration portal: a resource for rat somatosensory cortex. Front Neural Circuits. 2015. doi:10.3389/fncir.2015.00044
[7] Fiath, et al. Recording site placement on planar silicon-based probes affects signal quality in acute neuronal recordings. Scientific Reports. 2021. Doi: 10.1038/s41598-021-81127-5
[8] IMEC & Neuropixels. Neuropixels 1.0 – High resolution fully integrated silicon neural probe: Spec sheet [Internet]. Available from: https://www.neuropixels.org/_files/ugd/328966_c5e4d31e8a974962b5eb8ec975408c9f.pdf
[9] Fiath, et al. Recording site placement on planar silicon-based probes affects signal quality in acute neuronal recordings. Scientific Reports. 2021. Doi: 10.1038/s41598-021-81127-5

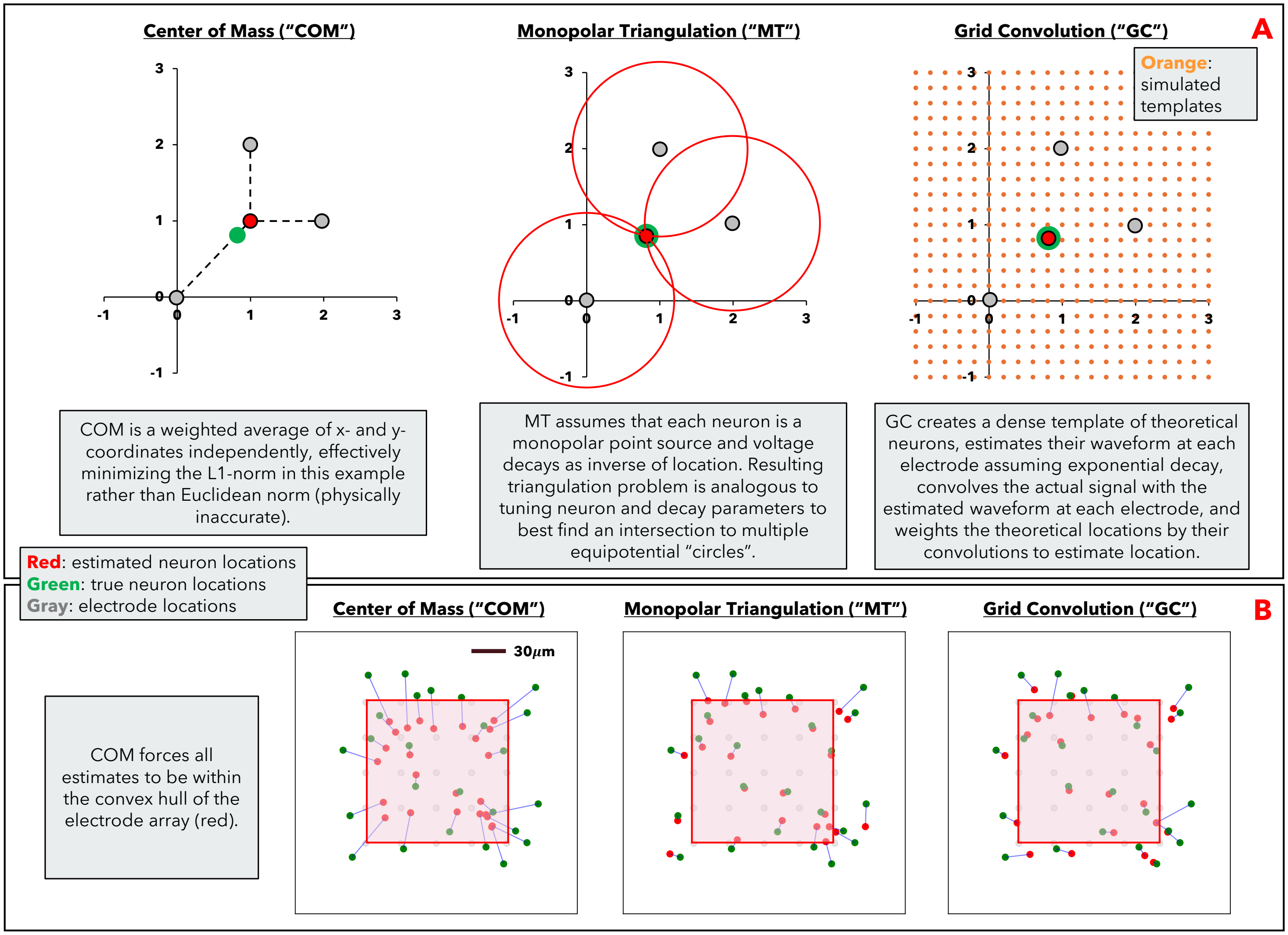

Fig 1. Limitations of center of mass localization. **A**: Visualization of localization algorithms for a point-source neuron equidistant to three electrodes located on a grid at (0,0), (1,2), and (2,1), where the electrodes will all receive the same signal strength from the neuron in ideal recording conditions (no noise or electrode degradation), given the neuron is located equidistant to all three electrodes. MT and GC are able to correctly estimate the "true" location while COM is not. **B**: Simulated electrophysiological data (MEArec), where green dots denote true neuron locations, red dots denote estimated neuron locations, and blue lines connect the true and estimated locations for a given neuron. Since COM is a weighted average of electrode locations, it forces all location estimates to be within the convex hull of the electrode array (all estimated neuron locations are within red square).

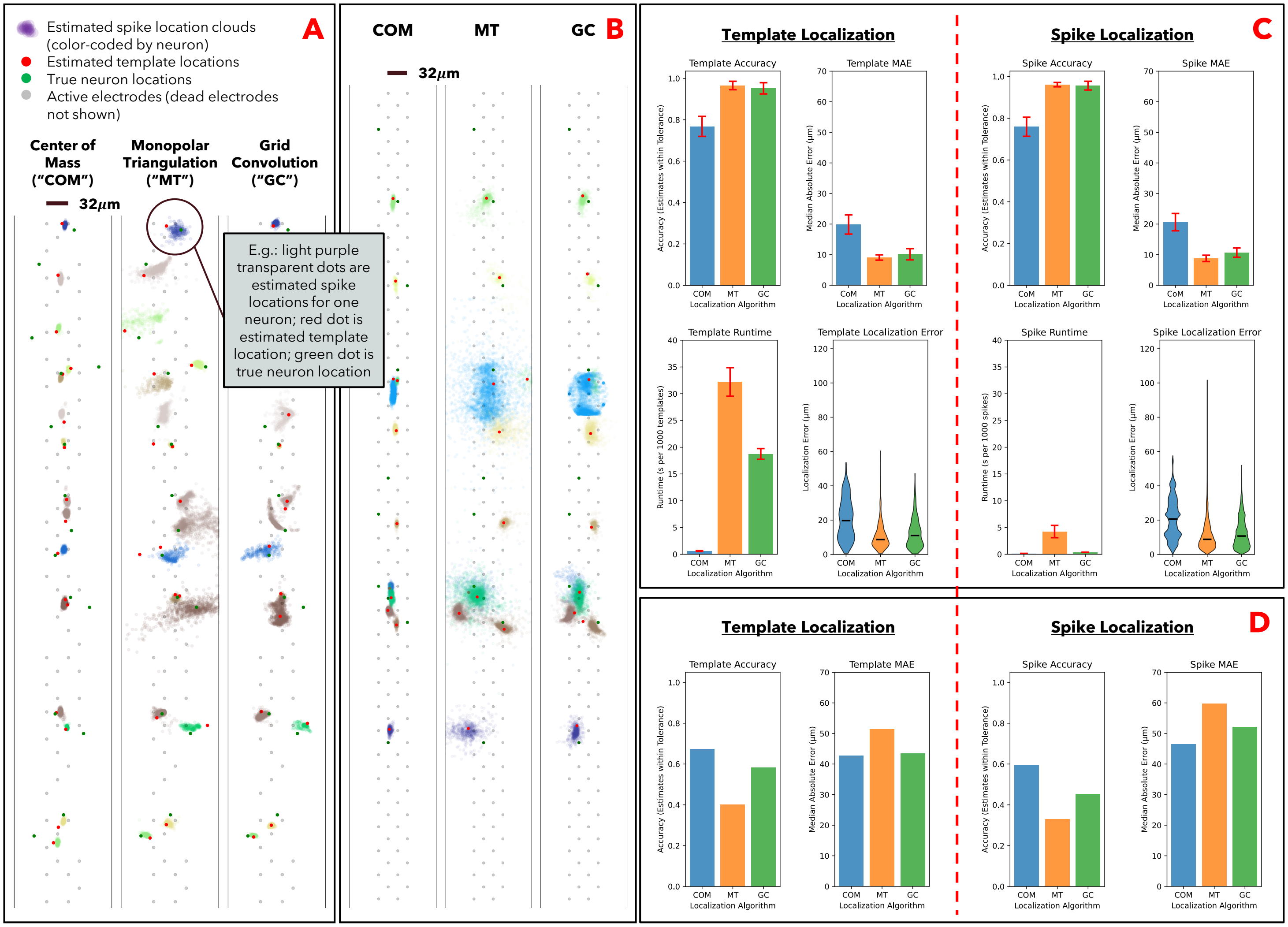

Fig 2. SSL algorithm performance with no electrode degradation. **A**: Visualization of template and spike localization estimates on simulated dataset (MEArec). The estimated spike locations are represented by translucent dot clouds, color-coded by their corresponding neuron. The estimated template location and true neuron locations are represented by solid red and green dots, respectively, and are not color-coded by neuron. The electrode array (shown excluding the "dead" electrodes, illustrating electrode degradation) is denoted by light gray dots. **B**: Visualization of template and spike localization estimates on experimental dataset (SPE-1). **C**: Performance metrics on simulated dataset, for both templates (left) and spikes (right). Performance is assessed as (i) percentage of estimates within 30μm of true locations (accuracy), (ii) speed of algorithm on all templates/spikes (runtime), and (iii) Euclidean distance between estimates and true location (median localization error and violin plot of individual localization errors). Bars represent mean metric across multiple simulations; error bars represent standard deviation of metric. Statistical significance was assessed using one-way ANOVA followed by Tukey's HSD post-hoc tests; all metrics exhibited significant differences (ANOVA $p < 0.05$). **D**. Performance metrics on experimental dataset, for both templates (left) and spikes (right). Performance is assessed using accuracy and runtime; since we have lower resolution of true locations in experimental data, we use a higher tolerance for accuracy (deemed correct if estimate within 50μm of estimated true location). We additionally do not include runtime, as the experimental neurons were recorded across different recording sessions (we concatenate onto one probe in Fig. 2B for presentation purposes), which does not allow for localization algorithm to amortize certain overhead costs across neurons. Bars represent metric across all experimental data.

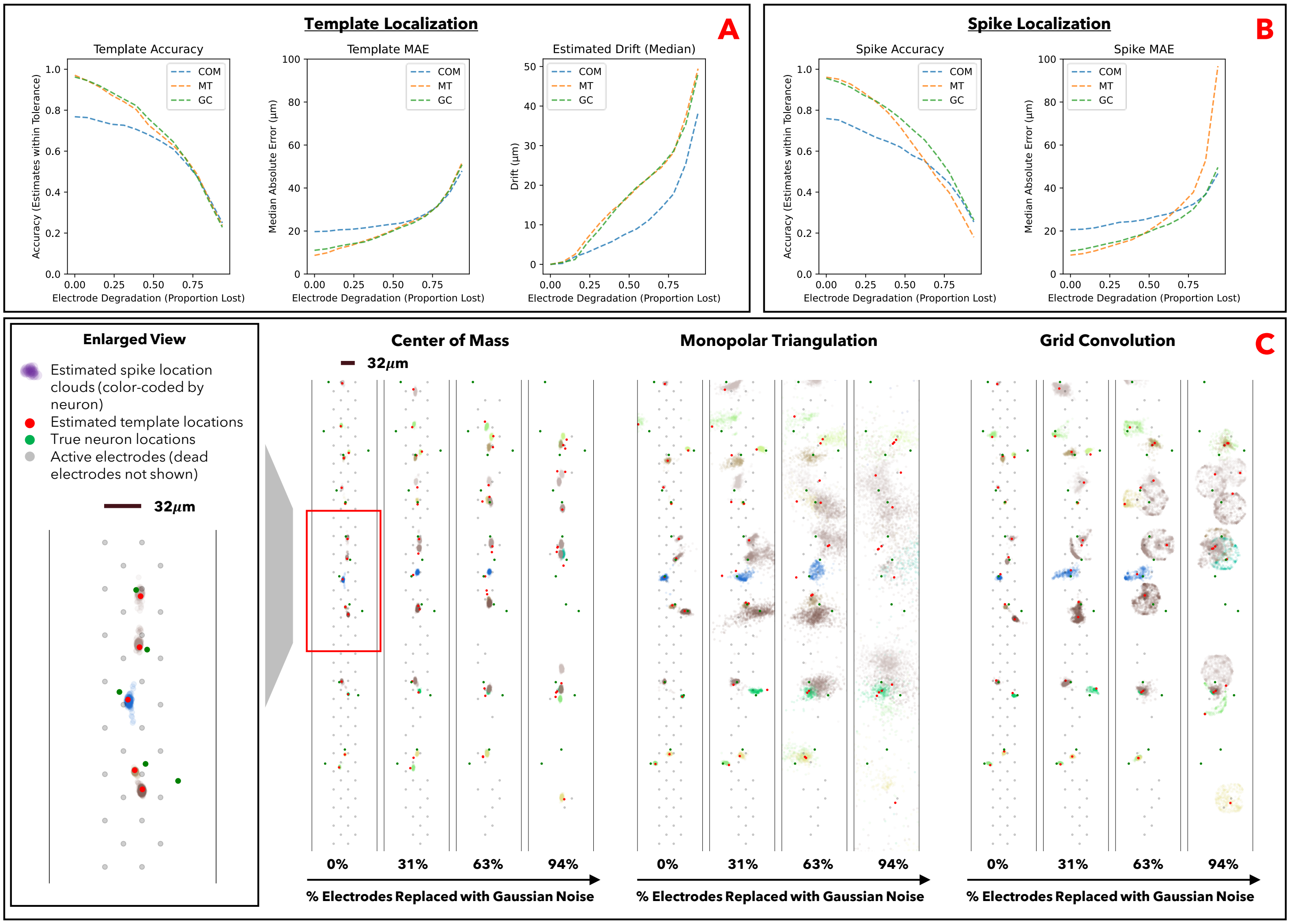

Fig 3. SSL algorithm robustness on simulated data (MEArec). **A**: Template localization performance metrics at varying levels of electrode degradation. Performance is assessed as (i) percentage of estimated locations within 30µm of true locations (accuracy), (ii) median Euclidean distance between estimated and true locations (localization error), and (iii) estimated drift from original location. Estimated drift is the Euclidean distance between the estimated template location without degradation v. the estimated template location with degradation; i.e., a measure of how unstable the localization algorithm is against degradation. **B**: Spike localization performance metrics at varying levels of electrode degradation. Performance is assessed via accuracy and localization error. C: Visualization of template and spike localization estimates at varying levels of electrode degradation. Left panel is zoomed-in view of visualization outlined in red.

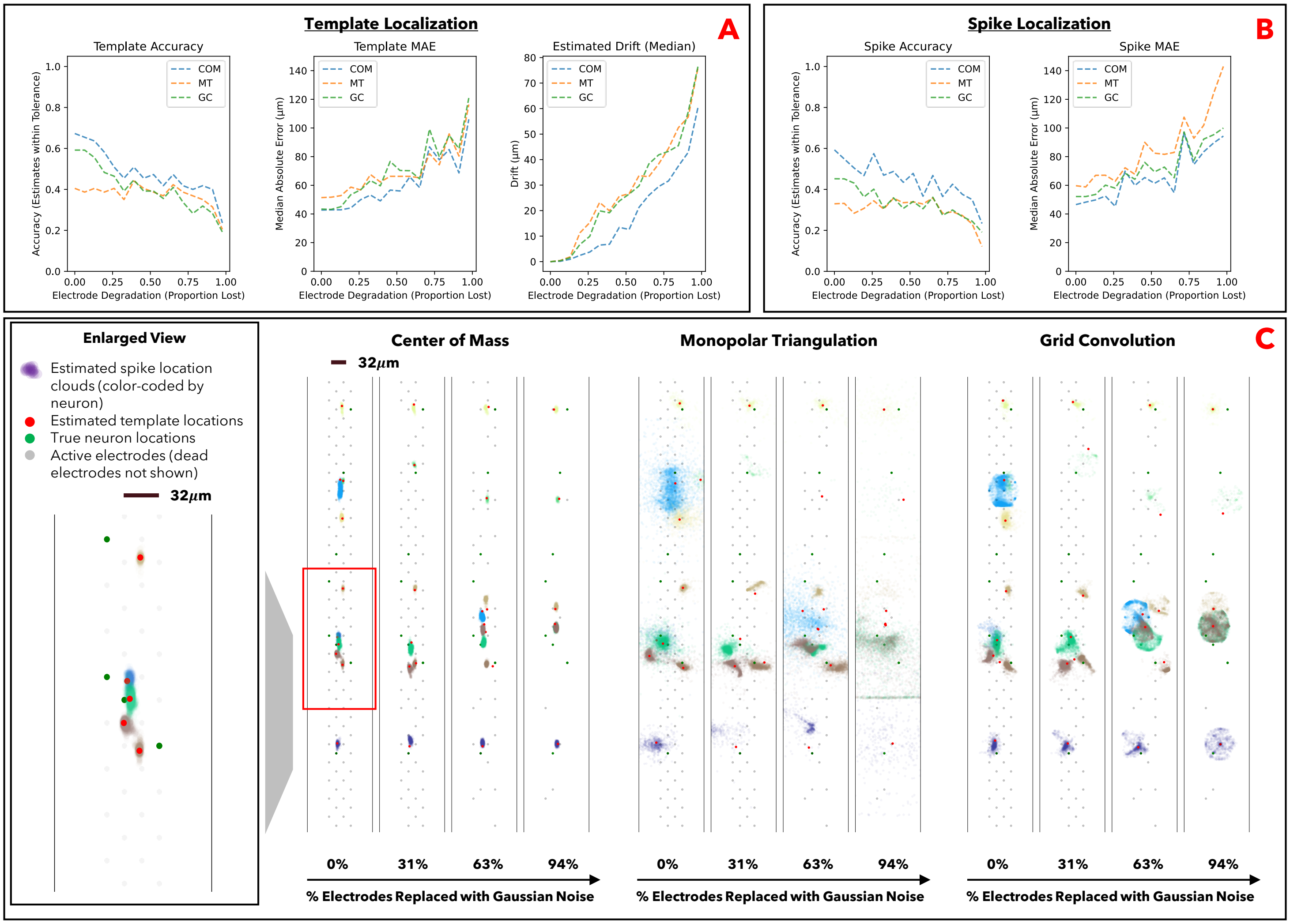

Fig 4. SSL algorithm robustness on experimental data (SPE-1). **A**: Template localization performance metrics at varying levels of electrode degradation (measured as the percentage of electrodes replaced with Gaussian noise). Performance is assessed as (i) percentage of estimated locations within 50µm of true locations (accuracy), (ii) median Euclidean distance between estimated and true locations (localization error), and (iii) estaimted drift from original location. Since we have lower resolution actual location in experimental data v. simulated data, we use a higher tolerance for accuracy v. simulated data (50µm v. 30µm). **B**: Spike localization performance metrics at varying levels of electrode degradation. Performance is assessed via accuracy and localization error. **C**: Visualization of template and spike localization estimates at varying levels of electrode degradation. Left panel is zoomed-in view of visualization outlined in red.
