# Supplementary material for: Benchmarking spike source localization algorithms in high density probes
Source: PLoS Comput Biol. 2026 Mar 16;22(3):e1014059. doi: 10.1371/journal.pcbi.1014059 (PMC13002180; doi:10.1371/journal.pcbi.1014059)
Supplement: Attachment — Submitted filename: Response to Reviewer (2026.02.18).pdf [file pcbi.1014059.s003.pdf]

Response to Reviewer #1 Comments:

General Comment: The authors have addressed the couple of large revisions requested. Namely, (1) on the geometry of the synthetic dataset electrodes raised by both reviewers, they regenerated and re-ran the algorithm on a synthetic dataset matching the electrode configurations of the experimental dataset. (2) They addressed the comment about the noise levels on synthetic data and set levels more in line with experimental data (note that to be thorough, the noise should not be white or gaussian, but have a non-flat PSD like pink noise, yet this is perfectly publishable in this state and I should have raised it on the original review).

They clarified the pre-processing by adding to the methods and removed claims about run-time. There remains only minor revisions and a suggestion.

Response to General Comment: We sincerely thank the reviewer for their thorough reading of the revised manuscript and encouraging comments. We are happy to present some final clarifications and improvements prior to publication. We address point by point the remaining revisions and suggestion below.

Comment #1: On the data sharing, I think my comment has been misunderstood. As of now, someone wanting to reproduce those results has to download terabytes of data and re-run a non-trivial pre-processing pipeline. I was suggesting to provide spike trains and average waveforms, with the corresponding localisation results. For 11 cells this would hardly be more than a few tens of megabytes, and not 4 terabytes as the authors have commented. Yet it would make the dataset much more accessible to anyone wanting to play with localisation algorithms.

Going further, providing 256 - 512 individual spike waveforms per cell would barely reach hundred megabytes, and again prove a much more approachable dataset to anyone wanting a quick look in the matter instead of wading through the non-trivial pre-processing.

Response to Comment #1: We thank the reviewer for this clarification. We fully agree that providing an accessible intermediate dataset is important towards our goal of providing a reproducible and re-usable benchmark for spike source localization algorithms.

To this end, we have reformatted our intermediate waveform datatypes to remove dependencies from the recording data (which was previously a feature of the data type used for the simulated MEArec data and inflated the size of the intermediate data). We have uploaded the full intermediate datasets (which include spike trains, template waveforms, and spike waveforms) to the github repository.

If the user were additionally interested in the full recording data, they are able to generate the MEArec recording data fully reproducibly using our seeded pipeline, and the SPE-1 dataset is publicly available. We have updated the manuscript accordingly.

Revisions to Manuscript: We have included the following in "Supporting Information":

All analysis scripts and supporting functions used in this study are available at https://github.com/haozhao1996/Spike-Localization-Algorithms, where we have additionally included the intermediate data including spike trains, waveforms, and localization values for both the simulated and experimental datasets. The code is released under the MIT License, permitting reuse, modification, and distribution with attribution. The simulated dataset is generated using MEArec (generation pipeline included in repository, and source code publicly available), and the experimental dataset is SPE-1 (publicly available).

Comment #2: l. 94 I wouldn't qualify a 300-3000 Hz bandpass filter standard, as the highcut frequency is unusual: it is standard in kilosort to use a 3d order 300 Hz highpass applied forward and backward (making it a sixth order zero-phase filter). Remove "standard" and state the filter order and if it is causal or zero-phase.

Response to Comment #2: We thank the reviewer for this insight. Our preprocessing pipeline utilizes a fifth-order, causal (Butterworth) bandpass filter from 300 – 3,000 Hz. We have clarified in the manuscript accordingly and removed the "standard" terminology.

Revisions to Manuscript: We include the following in "Data and Methods":

A spike sorting pre-processing pipeline for denoising and anomalous channel detection was applied to both the simulated and experimental datasets. A 300 - 3,000 Hz Butterworth bandpass filter (fifth-order, causal filter) was applied to the raw recordings, followed by common median referencing. We then perform anomalous channel detection using the established IBL protocol, detecting anomalous channels via coherence (i.e., adjacent channels within brain tissue should have a high degree of signal correlation) and power distribution (i.e., healthy channels should have a normal power distribution across frequencies).

Comment #3: l. 60 Aggregation: which one? It could be a count, the min, max, the average or median. I suspect it is the median or average but worth being more precise!

Response to Comment #3: We thank the reviewer for pointing this out. The templates are formed using the median across spike waveforms, which we have clarified in the manuscript.

Revisions to Manuscript: We include the following in "Data and Methods":

From the simulated dataset, we know the ground-truth neuron locations and spike trains used to generate the recording data, and we use these ground-truth spike trains to extract spike waveforms and template waveforms (constructed using the median across spike waveforms for a given neuron) at each recording electrode.

Comment #4: For the suggestion, I still think it would be nice to have some grid search of the localisation parameters. Here the authors are relying on spikeinterface default parameters, and dismissed the recommendation of exploring meta-parameters as the study being only within the scope of this standard use. Yet I would enjoin the authors to be a bit more careful about assuming adequacy of those default parameters, and to be frank, I would also enjoin them to be more curious. For example, what if your results exhibit a large sensitivity to those parameters ? Not only that would diminish some of this study conclusions, but that would also mean optimizing the parameter search may be an interesting avenue for improving the localisation method.

Response to Comment #4: We thank the reviewer for the comment, and as suggested have performed a parameter search for monopolar triangulation and grid convolution. We agree with the reviewer that these algorithms are more impacted by parameter choice (v. center of mass), as are many algorithms related to spike sorting or probe drift correction.

For monopolar triangulation, we examine the parameters "radius_um" (denoting the radius around the peak channel where signal is considered in optimization) and "max_distance_um" (denoting the max distance from the initial COM estimate considered as solution to optimization). In our parameter search, we evaluate parameters order of 2 lower and higher than the default values. For the template localization problem, the default parameters are 75 µm for radius_um and 1,000 µm for max_distance_um; we evaluate radius_um of [37.5, 75, 150] and max_distance_um of [500, 1000, 2000]. For the spike localization problem, the default parameters are 75 µm for radius_um and 150 µm for max_distance_um; we evaluate radius_um of [37.5, 75, 150] and max_distance_um of [75, 150, 300].

For grid convolution, we examine the parameters "radius_um" (denoting the radius around the peak channel where signal is considered in optimization) and "percentile" (denoting the percentage (in range [0, 100]) of the best scalar products used in weighted average). Similar to parameter search for MT, we evaluate parameters order of 2 lower and higher than the default values. For both the template and spike localization problems, the default parameters are 40 µm for radius_um and 5 for percentile; we evaluate radius_um of [20, 40, 80] and percentile of [2.5, 5, 10].

We have presented the parameter search results for the median spike localization error in a new Figure 5 of the manuscript (Fig 5. Impact of localization parameters on median spike localization error), also presented below. We highlight two interesting observations.

First, we observe that the study conclusions are robust to parameter choice, which provides additional confidence in these findings. Across parameter choices in the simulated dataset, we continue to see that MT and GC perform better in the lower degradation conditions, and GC and COM perform better in higher degradation conditions. In the experimental dataset, we continue to see that COM performs marginally better than GC, and substantially better than MT across degradation conditions.

Second, we observe that in each grid, the "upper left" represents higher regularization and the "lower right" represents lower regularization. For MT, the top left parameter set considers a smaller local radius of electrode signals (i.e., considers fewer, stronger signals) and limits the solutions to a smaller solution set. For GC, the top left parameter set also considers a smaller local radius of electrode signals and uses a smaller percentage of convolution results (i.e., fewer, stronger signals) in the weighted average. Under the lower degradation conditions in the simulated dataset, the less regularized parameter sets perform better than the more regularized parameter sets ("lower right" has lower errors than "upper left"); as electrode degradation increases, this observation "flips" and the more regularized parameters generally perform better than the less regularized parameters. This trend is also evident in the experimental dataset, though not as clearly indicated as the experimental dataset is inherently noisier (towards higher degradation conditions, we also see "lower right" performing worse in experimental dataset). This is consistent with our findings on how COM and GC perform better than MT under noisier conditions; when degradation and noise increase, the increased regularization is beneficial to accurate localization.

Revisions to Manuscript: We have included a new Figure 5, which we append at the end of this response letter. We have additionally included the following in "Results":

We note that MT and GC have a meaningful reliance on parameters regulating algorithm performance, including parameters regulating which channel signals are evaluated and the candidate window for solutions. While our results are based on the default values of these parameters in the SpikeInterface implementation, we have additionally performed a grid search on how different parameter sets may affect our study conclusions (Fig. 5). We note that that the study conclusions are robust to parameter choice. Across parameter choices in the simulated dataset, we continue to see that MT and GC perform better in the lower degradation conditions, and GC and COM perform better in higher degradation conditions. In the experimental dataset, we continue to see that COM performs marginally better than GC, and substantially better than MT across degradation conditions. We additionally observe that in each grid, the "upper left" represents higher regularization and the "lower right" represents lower regularization. For MT, the top left parameter set considers a smaller local radius of electrode signals (i.e., considers fewer, stronger signals) and limits the solutions to a smaller solution set. For GC, the top left parameter set also considers a smaller local radius of electrode signals and uses a smaller percentage of convolution results (i.e., fewer, stronger signals) in the weighted average. Under the lower degradation conditions in the simulated dataset, the less regularized parameter sets perform better than the more regularized parameter sets ("lower right" has lower errors than "upper left"); as electrode degradation increases, this observation "flips" and the more regularized parameters generally perform better than the less regularized parameters. This trend is also evident in the experimental dataset, though not as clearly indicated as the experimental dataset is inherently noisier (towards higher degradation conditions, we also see "lower right" performing worse in experimental dataset). This is consistent with our findings on how COM and GC perform better than MT under noisier conditions; when degradation and noise increase, the increased regularization is beneficial to accurate localization.

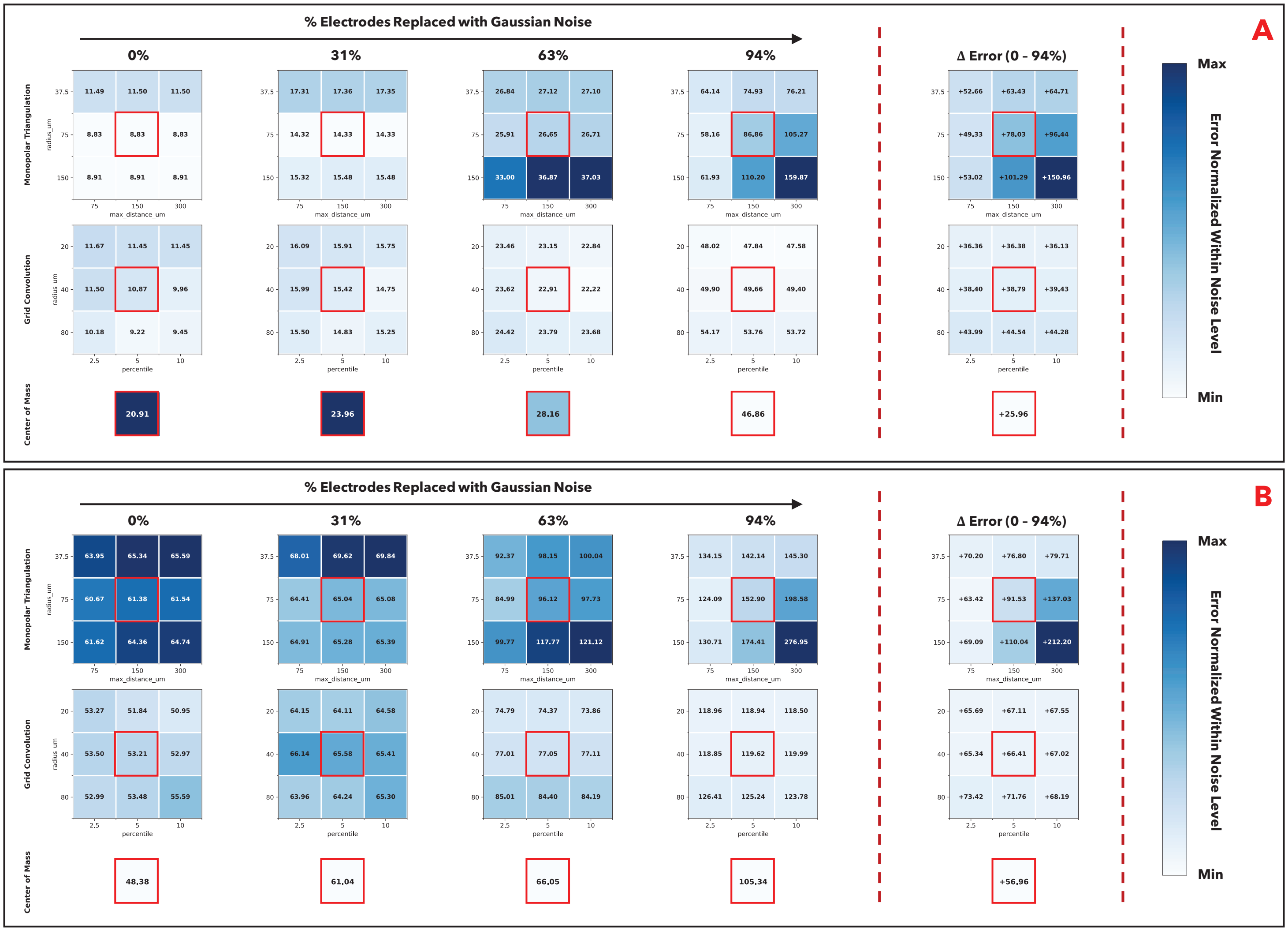

Fig 5. Impact of localization parameters on median spike localization error. For MT, we examine the parameters "radius_um" (denoting the radius around the peak channel where signal is considered in optimization) and "max_distance_um" (denoting the max distance from the COM initial estimate considered as a solution to optimization). For GC, we examine the parameters "radius_um" (similarly denoting radius around the peak channel where signal is considered in optimization) and "percentile" (denoting percentage of the best scalar products used to estimate location). For COM, the parameters have less meaningful impact on algorithm performance and so we present the median spike localization error under default parameters. Median error using the default parameters are marked in red boxes, and we examine parameters an order of two lower and higher than the default parameters. In the first four columns, we show the progression of the median localization error as electrode degradation increases, and the colors denoting magnitude of error are normalized within the individual electrode degradation level but across the three localization methods (i.e., normalized within the "column"). We additionally show the change in localization error from 0% to 94% degradation for each method and parameter set in the fifth column, to indicate how much electrode degradation impacts performance. **A**: Results on the simulated dataset (MEArec). **B**: Results on the experimental dataset (SPE-1).

<u>Response to Reviewer #2 Comments:</u>

General Comment: The authors have responded well to all of my concerns, and I have no further comments. I recommend that the manuscript is accepted.

Response to General Comment: We thank the reviewer for their remarks. Their review has helped in substantially improving this paper, and we are grateful for their suggestions throughout the review process and recommendation of acceptance.
